# Tapered wedge stems decrease early postoperative subsidence following cementless total hip arthroplasty in Dorr type C femurs compared to fit-and-fill stems

**DOI:** 10.1186/s13018-022-03111-7

**Published:** 2022-04-10

**Authors:** Satoshi Ikemura, Goro Motomura, Satoshi Hamai, Masanori Fujii, Shinya Kawahara, Taishi Sato, Daisuke Hara, Kyohei Shiomoto, Yasuharu Nakashima

**Affiliations:** Department of Orthopaedic Surgery, Graduate School of Medical Sciences, Kyushu University, 3-1-1 Maidashi, Higashi-ku, Fukuoka 812-8582 Japan

**Keywords:** Cementless total hip arthroplasty, Stem subsidence, Dorr type C, Tapered wedge stem, Fit-and-fill stem

## Abstract

**Background:**

To compare the degree of stem subsidence between two different femoral component designs and to determine the risk factors associated with stem subsidence after cementless total hip arthroplasty (THA) in Dorr type C femurs.

**Methods:**

We retrospectively reviewed 104 consecutive hips in 100 patients with Dorr type C proximal femoral morphology who underwent primary cementless THA using a fit-and-fill stem or a tapered wedge femoral stem at our institution between January 2012 and June 2021. A fit-and-fill stem was used in 55 hips and a tapered wedge stem was used in 49 hips. Radiologically, the distance between the apex of the major trochanter and the stem shoulder were measured at three different time points (immediately [0W], one week [1W], and six weeks [6W] after surgery) and the degrees of stem subsidence were assessed by comparing the distance between 0 and 1W, 1W and 6W, and 0W and 6W, respectively.

**Results:**

The mean degree of stem subsidence (0W vs. 1W) was 0.24 mm (standard deviation [SD] 0.36) in the fit-and-fill stem group, and 0.23 mm (SD 0.41) in the tapered wedge stem group. There was no significant difference between the two groups (*P* = 0.4862). However, the mean degrees of subsidence were significantly higher in the fit-and-fill stem group (1W vs. 6W, 0.38 mm [SD 0.68]; 0W vs. 6W, 0.65 mm [SD 0.87]) than in the tapered wedge stem group (1W vs. 6W, 0.16 mm [SD 0.32]; 0W vs. 6W, 0.24 mm [SD 0.38]) (*P* < 0.05 for both). In addition, the rates of > 3 mm subsidence (in which instability can be observed) were 18.2% (10 of 55 hips) and 2.0% (1 of 49 hips), respectively. There was also a significant difference between the two stems (*P* = 0.0091). Multivariate analysis demonstrated that fit-and-fill stem was a risk factor for > 3 mm subsidence after THA in Dorr type C femurs (*P* = 0.0050).

**Conclusion:**

Our findings suggest that the tapered wedge stem is more suitable for Dorr type C femurs than the fit-and-fill stem to avoid early postoperative subsidence in cementless THA.

## Background

Total hip arthroplasty (THA) is one of the most successful surgeries of the twentieth century to alleviate pain and restore joint function in patients with end-stage hip arthritis [1]. The number of surgeries performed each year and the degree of durability have been increasing because of the increasing average life expectancy [2–4]. However, stem subsidence is a known cause of early failure of surgery, in addition to periprosthetic femoral fracture (PFF), instability, and infection [5–7]. Maximum stem subsidence is observed within the first six to eight weeks after cementless THA [8–10]. It can lead to unequal leg length, decreased hip stability, and implant failure. While stem subsidence of up to 3 mm is considered acceptable, migration above 5 mm is associated with early failure due to the instability of the hip joint [11, 12]. Several published studies have revealed the relationship between stem subsidence and survivorship [13–17]. Streit et al. [17] demonstrated the survivorship would drop to 29% if the subsidence was more than 2.7 mm.

A number of studies have reported risk factors for stem subsidence after cementless THA such as male sex, obesity, osteoporosis, and Dorr type C femoral morphology [18–20]. In 1993, Dorr et al. divided the morphology of the proximal femur into three types: type A (champagne-flute), type B (funnel type), and type C (stovepipe) [21]. Dorr type C femoral morphology is characterized by thin cortices, which are found predominantly in elderly patients with osteoporosis. It is believed that patients with Dorr type C femurs may lack structural support to achieve initial stability for the tapered, cementless stem, consequently resulting in subsidence, early loosening, and failure of the prosthesis [22, 23]. In contrast, several studies reported good clinical and radiological results of mid- to long-term follow-up periods without stem subsidence [24, 25]. It is still controversial as to what type of cementless stem is optimal for Dorr type C, since no studies have compared different types of stems in terms of stem subsidence after cementless THA while focusing on Dorr type C femurs. Therefore, the purposes of this study were to compare the degree of stem subsidence between two different femoral component designs and to determine the risk factors associated with stem subsidence after cementless THA in Dorr type C femurs.

## Methods

**Fig. 1:**
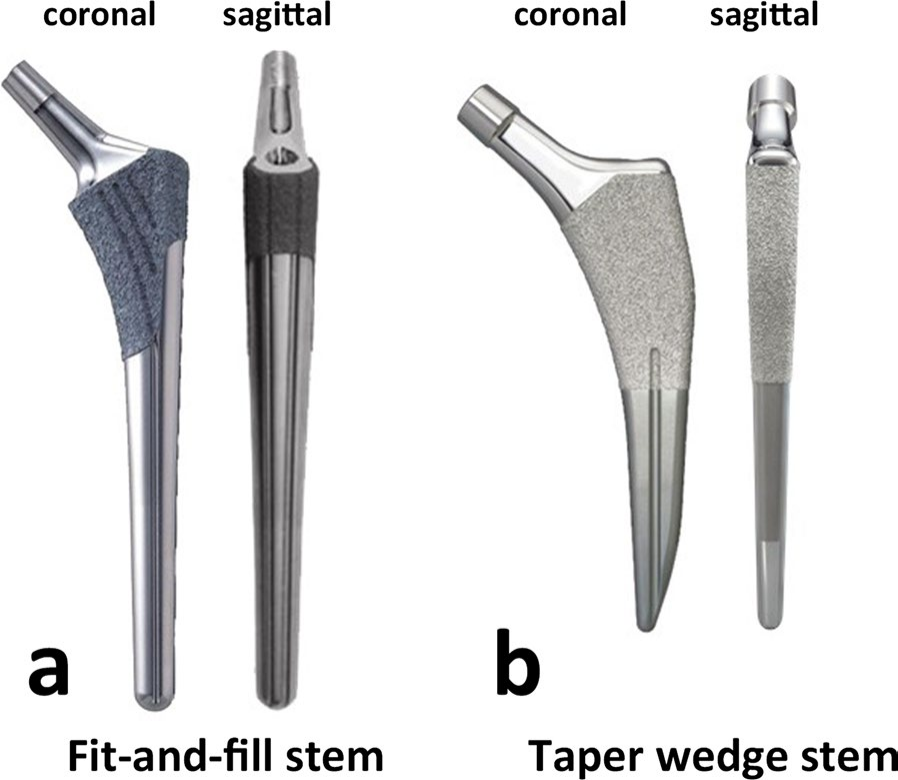
**a** Coronal and sagittal profile of PerFix910 (Kyocera, Kyoto, Japan) femoral component. **b** Coronal and sagittal profile of Initia (Kyocera, Kyoto, Japan) femoral component

This study was approved by our institutional review board. The need for informed consent was waived due to the retrospective and anonymous study design. Cementless THA using posterolateral approach was performed in 107 hips of 103 patients with Dorr type C proximal femoral morphology at our institution between January 2012 and June 2021. Five experienced hip surgeons performed the operation, each with experience of > 50 THAs/year. A fit-and-fill stem (Fig. 1a; PerFix910, Kyocera, Kyoto, Japan) was used in 57 hips, and a tapered wedge stem (Fig. 1b; Initia, Kyocera, Kyoto, Japan) was used in 50 hips. At our institution, a conventional fit-and-fill stem has been used for the cementless THA. However, we have been using a modern tapered wedge stem for various types of femoral morphology in THA since August 2017. Therefore, the choice of stem type was made based on historical usage. In the fit-and-fill stem group only, PFF occurred in 2 of 57 hips (3.5%). One hip had an intraoperative calcar fracture and was treated with circumference wiring, and the other had postoperative fracture at the level of the greater trochanter (Vancouver type AG) 8 days after surgery [26]. In the tapered wedge stem group, dislocation of the hip joint was observed in one hip 3 weeks after the surgery. These three hips were excluded from this study. Neither superficial nor deep infection were observed in either stem groups. Therefore, we retrospectively reviewed 104 consecutive hips in 100 patients with Dorr type C proximal femoral morphology. The patients included 16 males and 84 females with a mean age at surgery of 67 years (range 32–87). The primary reason for surgery was osteoarthritis (OA) in 95 hips, osteonecrosis of the femoral head (ONFH) in 7 hips, and rheumatoid arthritis (RA) in 2 hips. The mean body mass index (BMI) was 24.1 kg/m^2^. Full weight-bearing walking was started the day after surgery in all 100 patients (104 hips). A clinical assessment based on the Harris hip score (HHS) was made pre-and post-operatively [27].

**Fig. 2:**
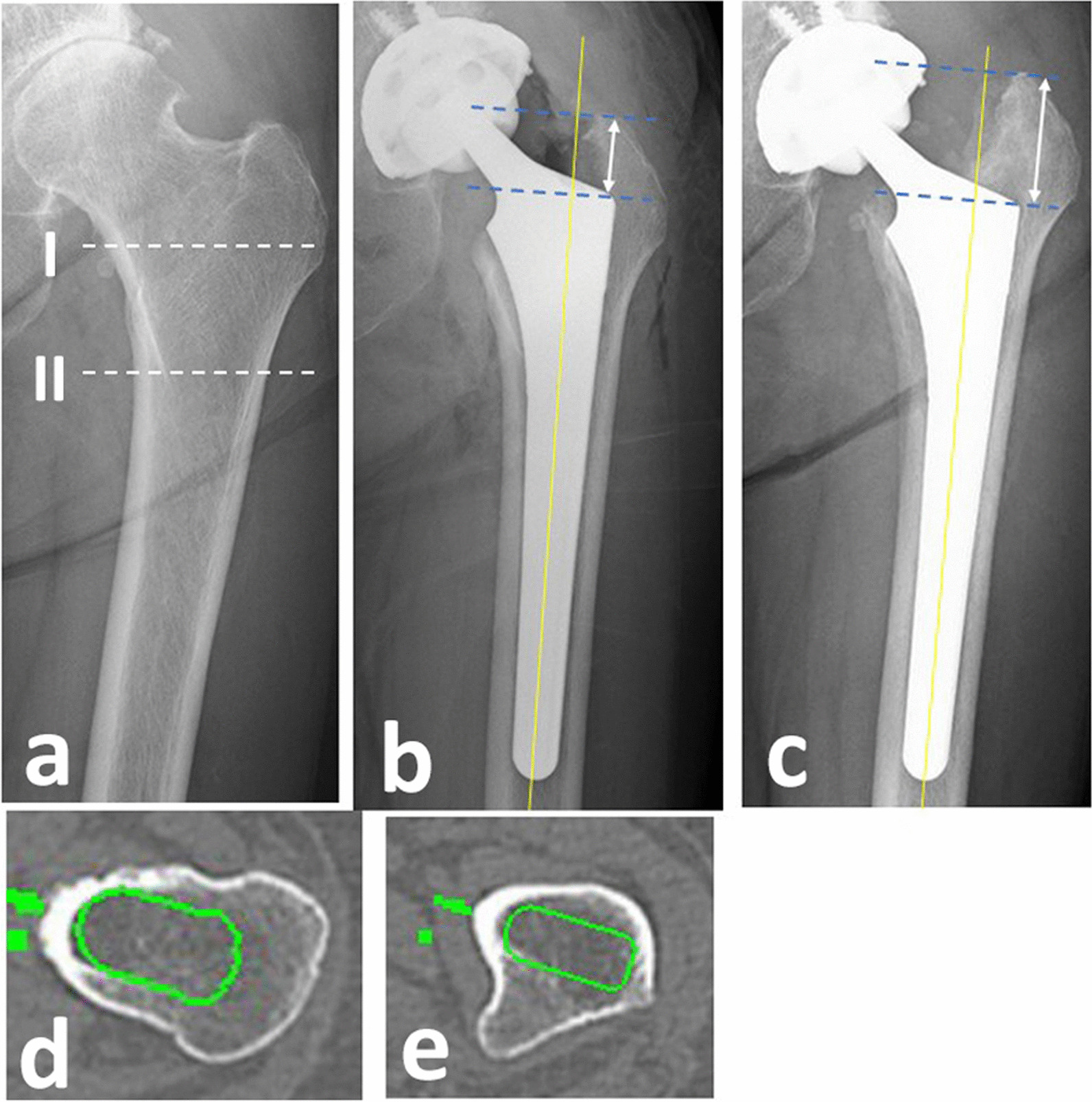
**a** Left hip joint radiograph of a 78-year-old female with advanced osteoarthritis. (I) The level of fixation of fit-and-fill stem, (II) The level of fixation of tapered wedge stem. **b** Patient was treated by cementless total hip arthroplasty (THA) using fit-and-fill stem. **c** The degree of stem subsidence is 8.2 mm 6 weeks after the surgery. **d** Preoperative three-dimensional (3D) planning (axial plane) for fit-and-fill stem (3D template, Kyocera, Kyoto, Japan) at the level of I in **a**. **e** Preoperative 3D planning (axial plane) for tapered wedge stem at the level of II in **a**

Postoperative radiographs were assessed by two observers (S.I. and K.S.) who are orthopedic surgeons with extensive diagnostic imaging experience, using digital imaging system (Synapse Digital Imaging System, Fujifilm Medical Imaging, Tokyo, Japan), and only mean values were used for statistical analysis. Subsidence was defined as a femoral stem distalization in reference to the major trochanter, according to a previous report [20]. The distance between the apex of the major trochanter and the stem shoulder was measured at three different time points (immediately [0W], one week [1W], and six weeks [6W] after surgery) and the degrees of stem subsidence were assessed by comparing the distance at each time point (0W vs. 1W, 1W vs. 6W, and 0W vs. 6W) in both stems (Fig. 2). We standardized the rotation of the lower limbs on anteroposterior radiographs by positioning both patellae in the exact frontal position throughout the radiological examination. After measurement of the stem subsidence from 0 to 6W, all 104 hips were divided into two subgroups (> 3 mm subsidence positive or negative) based on the degree of subsidence [11, 12].

Statistical analyses were performed using the JMP Ver. 9.0.1 software (SAS Institute Inc., Cary, NC, USA). All data were tested for normality using the Shapiroe-Wilk test and for homoscedasticity using the Levene's test. Normally distributed variables (age, BMI, and degree of stem subsidence) were compared between the fit-and-fill stem and tapered wedge stem groups using a Student’s *t*-test. The non-normally distributed variable (HHS) was evaluated using the Wilcoxon rank-sum test. Fisher’s exact probability test was used to compare the sex, reasons for surgery, and the incidence of PFF between the two groups (fit-and-fill stem vs. tapered wedge stem and > 3 mm subsidence positive vs. negative). Multivariate analysis was performed to identify parameters associated with > 3 mm stem subsidence from 0 to 6W after THA using a stepwise logistic regression with variable selection (*P* < 0.2). *P* values < 0.05 were considered statistically significant.

## Results

The detailed clinical findings of all patients are shown in Table 1. The patients in the fit-and-fill stem group included 5 males and 50 females with a mean age at surgery of 67.9 years (standard deviation [SD] 10.5) Patients in the tapered wedge stem group included 11 males and 38 females with a mean age of 65.3 years (SD 13.1). There were no significant differences in sex and age between the two groups (*P* = 0.1003 and 0.2605, respectively). The mean BMI was 24.0 kg/m^2^ (SD 4.0) in the fit-and-fill stem group, and 24.1 kg/m^2^ (SD 4.4) in the tapered wedge stem group. There was no significant difference between the two groups (*P* = 0.3426). Reasons for surgery included: 49 hips, OA; 4 hips, ONFH; and 2 hips, RA in the fit-and-fill group; and 46 hips, OA; and 3 hips, ONFH in the tapered wedge stem group. There was also no significant difference between the two groups (*P* = 0.4950). Clinically, the mean postoperative (6W) HHS (fit-and-fill, 84.2 [SD 8.9]; tapered wedge, 85.1 [SD 9.2]) was significantly improved compared to the mean preoperative HHS (fit-and-fill, 53.5 [SD 19.6]; tapered wedge, 59.1 [SD 17.6]) (*P* < 0.0001 for both groups).

**Table 1:** Clinical and imaging findings between fit-and-fill stem and tapered wedge stem groups in Dorr type C femurs

	Fit-and-fill stem group (*N* = 55)	Tapered wedge stem group (*N* = 49)	*P* values
Sex (male:female)	5:50	11:38	0.1003
Age (years)	67.9 ± 10.5	65.3 ± 13.1	0.2605
BMI (kg/m^2^)	24.0 ± 4.0	24.1 ± 4.4	0.3426
Diagnosis			0.4950
OA	49	46	
Other (ONFH or RA)	6	3	
HHS			
Preoperative	53.5 ± 19.6	59.1 ± 17.6	0.1320
Postoperative (6W)	84.2 ± 8.9	85.1 ± 9.2	0.6191
*Subsidence (mm)*			
Duration from			
0–1W	0.24 ± 0.36	0.23 ± 0.41	0.4862
1–6W	0.38 ± 0.68	0.16 ± 0.32	0.0423*
0–6W	0.65 ± 0.87	0.24 ± 0.38	0.0438*
> 3 mm subsidence	10 (18.2%)	1 (2.0%)	0.0091*

Continuous variables are represented as mean ± standard deviation

*N* number, *BMI* body mass index, *OA* osteoarthritis, *ONFH* osteonecrosis of the femoral head, *RA* rheumatoid arthritis, *HHS* Harris hip score, *0W* immediate after surgery, *1W* One week after the surgery, *6W* Six weeks after surgery

*P < 0.05 indicates significance

The mean degrees of stem subsidence from 1 to 6W and 0W to 6W were significantly higher in the fit-and-fill stem group than in the tapered wedge stem group (Table 1). The rate of > 3 mm subsidence was 18.2% (10 of 55 hips) in the fit-and-fill stem group (Fig. 2), and 2.0% (1 of 49 hips) in the tapered wedge stem group. There was a significant difference between the two groups.

Eleven hips were categorized as the > 3 mm stem subsidence-positive group and 93 as the − negative group (Table 2). Multivariate analysis demonstrated that type of cementless stem was independently associated with > 3 mm subsidence after THA in Dorr type C femurs (Table 3, *P* = 0.0050).

**Table 2:** Univariate analysis of > 3 mm stem subsidence-positive and − negative groups in Dorr type C femurs

	> 3 mm subsidence-positive (*N* = 11)	> 3 mm subsidence-negative (*N* = 93)	*P* values
Sex (male:female)	1:10	15:78	0.9999
Age (years)	71.9 ± 9.5	66.1 ± 11.9	0.1229
BMI (kg/m^2^)	23.7 ± 2.1	24.1 ± 4.4	0.7554
Diagnosis			0.2422
OA	9	86	
Other (ONFH or RA)	2	7	
Stem			0.0091*
Fit-and-fill	10	45	
Tapered wedge	1	48	
HHS			
Preoperative	58.8 ± 17.1	55.8 ± 19.1	0.6189
Postoperative (6W)	83.0 ± 10.8	84.8 ± 8.8	0.5225

Continuous variables are represented as mean ± standard deviation

*N*—number, *BMI*—body mass index, *OA*—osteoarthritis, *ONFH*—osteonecrosis of the femoral head, *RA*—rheumatoid arthritis, *HHS*—Harris hip score, *6W*—Six weeks after surgery

*P < 0.05 indicates significance

**Table 3:** Independent factors for > 3 mm stem subsidence after THA in Dorr type C femurs in multivariate analysis

	Odds ratio	95% Confidence interval	*P* value
Age	1.06	0.99–1.15	0.1255
Stem	10.37	1.84–195.62	0.0050*

*P < 0.05 indicates significance

## Discussion

Subsidence is a known complication of THA. Walker et al. found that subsidence of the femoral stem can predict clinical outcomes, and that decreased subsidence leads to better results [13]. Additionally, significant subsidence may contribute to postoperative instability and leg length discrepancy [11, 12]. A number of studies focusing on stem subsidence have been reported, since excessive subsidence can lead to early failure after surgery [5–12, 28]. Nevertheless, there have been few reports on subsidence comparing different types of stems. Grant et al. compared the subsidence of a cementless fit-and-fill stem with that of a cementless tapered wedge stem [28]. The mean degree of subsidence 4 weeks after THA was significantly lower in 65 patients treated with a tapered wedge stem (0.3 mm) than in 61 patients treated with a fit-and-fill stem (1.1 mm) [28], which was consistent with our results (taper wedge, 0.25 mm; fit-and-fill, 0.64 mm). However, their study did not analyze subsidence based on femoral morphology such as types of Dorr. Our study is the first to report that a tapered wedge stem is more suitable for immediate fixation than a fit-and-fill stem in Dorr type C femurs by comparing the degrees of stem subsidence between two different types of stems.

The advantages of cementless implantation including shorter operative times and lower risk of potential cardiopulmonary event, may favor the use of this stem [29, 30]. However, cemented stems are generally recommended for patients with Dorr type C femurs because of concerns about achieving immediate fixation and occurring of fracture around the cementless stem [29, 30]. An investigation by Pentlow and Heal showed a significantly higher subsidence rate of hydroxyapatite (HA)-coated cementless stem in traumatic patients who mostly had Dorr type C femurs [23]. Song et al. reported that Dorr type C femurs had a higher risk of subsidence when using uncemented collarless HA-coated stem [20]. In contrast, Reis et al. concluded in their investigation that no anatomic parameter could be detected as a predisposing factor for cementless stem subsidence [12]. Dalury et al. also reported that a proximally coated cementless stem was safely and successfully used for type C bone [24]. In the current study, the mean stem subsidence in the duration from immediate to 6 weeks after surgery was within 1 mm in both cementless stems, which supports previous reports in which cementless stems have been used safely for THA even for Dorr type C femurs. In addition, our study revealed for the first time that a tapered wedge stem is more suitable for immediate fixation in patients with bone fragility (Dorr type C femurs) than a fit-and-fill stem.

Regarding the lower degree of stem subsidence in the tapered wedge stem group than in the fit-and-fill stem group observed in our study, a recent comparative study mentioned that tapered wedge stems are designed to achieve more congruent cortical fit in the coronal plane and have demonstrated excellent long-term results of 25 years with a small 1.4% loosening rate [28, 31]. We consider that mediolateral cortical bone quality at the level of initial fixation in the tapered wedge stem (bellow the lesser trochanter) is better than that in the fit-and-fill stem (above the lesser trochanter) (Fig. 2) in the majority of Dorr type C femurs, which may be related to the differences in the degree of subsidence between the two groups in this study. Additionally, the tapered wedge stem may have the advantage of more accurate stem size prediction in preoperative three-dimensional (3D) planning, since the mediolateral cortical bone is clear compared to fit-and-fill stem (Fig. 2).

There were several limitations to the current study. First, this study was not prospective and randomized. Therefore, the precise effect of the type of stem on subsidence after THA in Dorr type C femurs should be further confirmed in a randomized controlled study. Second, the number of patients with more than 3 mm subsidence after THA was small, and a small sample size might have decreased the statistical reliability of our study. A multicenter study with a large sample size could provide more accurate and statistically reliable results. However, with a larger sample size, the number of surgeons would vary widely, which could make the analysis less precise. Third, this study evaluated stem subsidence only early (up to 6W) after surgery in both fit-and-fill and tapered wedge stems, although Dalury et al. reported that subsidence was apparent at 6 weeks and remained stable at an average of 6 years of follow-up [24]. Finally, the implants used in this study are not commonly used globally.

## Conclusion

Our findings suggest that cementless stems can be used safely for THA, even for Dorr type C femurs. In addition, the tapered wedge stem appears to be more suitable for Dorr type C femurs than the fit-and-fill stem to avoid early postoperative subsidence in cementless THA.

## Data Availability

The datasets used and/or analyzed during the current study are available from the corresponding author on reasonable request.

## Abbreviations

THA: Total hip arthroplasty
W: Week
SD: Standard deviation
BMI: Body mass index
PFF: Periprosthetic femoral fracture
OA: Osteoarthritis
ONFH: Osteonecrosis of the femoral head
RA: Rheumatoid arthritis
HHS: Harris hip score
HA: Hydroxyapatite
3D: Three-dimensional
